# Integrative multi-omics analysis reveals stress-specific molecular architectures in soybean under drought and rust infection

**DOI:** 10.1186/s12864-026-12673-3

**Published:** 2026-02-28

**Authors:** Gustavo Husein, Fernanda R. Castro-Moretti, Melina Prado, Lilian Amorim, Paulo Mazzafera, Javier Canales, Claudia B. Monteiro-Vitorello

**Affiliations:** 1 Department of Genetics, Luiz de Queiroz College of Agriculture, University of São Paulo, Piracicaba, Brazil; 2 Department of Plant Pathology and Nematology, Luiz de Queiroz College of Agriculture, University of São Paulo, Piracicaba, Brazil; 3 Department of Crop, Soil, and Environmental Sciences, University of Arkansas, Fayetteville, AR USA; 4 Department of Plant Biology, Institute of Biology, University of Campinas, Campinas, Brazil; 5 Institute of Biochemistry and Microbiology, Faculty of Sciences, Universidad Austral de Chile, Valdivia, Chile

**Keywords:** Glycine max, Abiotic and biotic stress, Transcriptomic, Metabolomic, Abscisic acid, Plant–pathogen interaction, Weighted Gene Co-expression Network Analysis (WGCNA), Copula Graphical Models (CGMs), Climate change

## Abstract

Asian soybean rust (ASR), caused by *Phakopsora pachyrhizi*, represents a major constraint to soybean cultivation, with yield losses approaching 90% in the absence of effective control strategies. When coupled with the increasing incidence of drought driven by climate change, the co-occurrence of these biotic and abiotic pressures imposes a complex challenge for crop resilience. In this study, we explored the molecular responses of soybean (*Glycine max*) to concurrent water limitation and ASR infection through an integrative analysis of transcriptomic and metabolomic datasets. To capture both linear and conditional relationships among molecular features, we employed Weighted Gene Co-expression Network Analysis (WGCNA) alongside Copula Graphical Models (CGMs). WGCNA identified 17 gene co-expression modules exhibiting significant correlations with 27 annotated metabolites. Among these, abscisic acid showed consistent associations with drought-responsive modules enriched in central metabolic pathways and transcription factors such as Dof and bHLH. In contrast, modules linked to fungal infection were correlated with dipeptides and D-galacturonic acid, implicating early defense signaling and cell wall remodeling. The CGM framework further revealed sparse, condition-specific networks of differentially expressed genes and metabolites directly associated with each stressor, including genes encoding a dirigent-like protein, pentatricopeptide repeat proteins, and a nucleoredoxin, as well as metabolites such as inosine, Epi-dihydrophaseic acid and 2-oxoadipic acid. Notably, no gene or metabolite was found to be directly responsive to both stresses, underscoring the modular and stress-specific architecture of soybean defense. These distinct, stress-specific modules operate in parallel during early responses to combined stress. Together, these results highlight a hierarchical regulatory structure and illustrate the value of combining correlation-based and dependency-driven models to identify candidate targets for multi-stress resilience breeding.

**Supplementary Information:**

The online version contains supplementary material available at 10.1186/s12864-026-12673-3.

## Introduction

Soybean (*Glycine max* (L.) Merr.) is one of the most important crops due to its high protein and oil content, supporting diverse applications in food, feed, and biofuel industries [1]. Despite its agronomic importance, soybean productivity is increasingly threatened by the occurrence of biotic and abiotic stresses. Asian soybean rust (ASR), caused by the biotrophic fungus *Phakopsora pachyrhizi*, is the most destructive soybean disease worldwide, capable of causing yield losses between 20% and 90% in the absence of chemical control, which to date remains the only efficient method to control this disease as there are no resistant genotypes [2]. Climate change is expected to exacerbate this threat by expanding the geographic distribution of pathogens and intensifying selection pressure on host resistance genes [3–6]. Simultaneously, water limitation stress is projected to increase in severity due to shifts in precipitation patterns and prolonged drought periods [7, 8]. The combined occurrence of abiotic and biotic stresses not only intensifies physiological damage, such as photosynthetic inhibition and premature defoliation [9], but also leads to complex and poorly understood interactions at the molecular level. These stress combinations are predicted to become more frequent and severe, yet their joint effects on plant development, metabolism, and defense remain insufficiently characterized [10–13]. Understanding the genetic and molecular basis of soybean responses to these concurrent stresses has therefore become a critical objective for improving crop resilience under current and future climatic scenarios [14–16].

Traditional approaches to elucidating molecular plant stress responses have typically relied on single-omics, focusing independently on transcriptomic or metabolomic data. While valuable, these strategies offer a fragmented view of molecular regulation, failing to capture how transcriptional and metabolic responses are temporally and functionally coordinated. Multi-omics frameworks have emerged as a powerful alternative, offering the possibility to map integrative regulatory circuits that span multiple biological layers [17, 18]. Among the available tools, weighted gene co-expression network analysis (WGCNA) is widely used to identify gene modules co-expressed under specific conditions and to relate them to phenotypic or metabolic traits [19–21]. However, as a correlation-based approach, WGCNA does not distinguish between direct and indirect associations, which may limit its capacity to resolve causally relevant components [22, 23]. Probabilistic graphical models represent an alternative strategy, providing a principled framework to infer conditional dependencies, which are associations that remain after accounting for the influence of all other measured variables [24, 25].

Among these strategies, Copula Graphical Models (CGMs) extend this concept to non-Gaussian and potentially non-linear dependencies, making them well suited to transcriptomic and metabolomic datasets that often violate assumptions of normality [26]. By estimating sparse precision matrices in a copula-transformed space, CGMs enable the identification of direct relationships that remain after accounting for all marginal dependencies, thereby revealing a network of true conditionally independent associations among genes, metabolites, and experimental factors [27]. Beyond their theoretical appeal, CGMs also offer practical advantages: they can handle missing data without the need for imputation, support the joint modeling of heterogeneous variable types, including both continuous and categorical features, and enable an exploratory, data-driven investigation of complex molecular systems [24, 28, 29]. This latter aspect is particularly relevant in multi-omics studies, as it mitigates biases introduced by prior variable selection and allows previously unanticipated interactions to emerge from the structure of the data itself. Despite their potential, CGMs remain underutilized in plant systems biology. While recent studies have applied CGMs to assess the impact of drought stress on maize and wheat yield [30], to dissect disease resistance in an interspecific raspberry population [31], and to identify the direct associations between dietary and epigenetic ageing in humans [32], their use in multi-omics integration, particularly under combined biotic and abiotic stress conditions in plants, remains virtually unexplored, and to our knowledge, this study represents the first application of CGMs in this context.

Building on previous single-omic studies of soybean responses to water limitation and *P. pachyrhizi* infection [33, 34], the present work represents a re-analysis by applying an integrative multi-omics strategy that combines transcriptomic and metabolomic profiling under ungal infection and water-limiting conditions. Using both WGCNA and CGM, we investigate whether the plant’s molecular response architecture is modular, stress-specific, or convergent under simultaneous stress exposure. This dual-framework approach enables the delineation of co-regulated gene–metabolite modules, the identification of direct associations relevant to each treatment condition, and the prioritization of candidate molecular components for future functional validation. Our findings provide deeper insights into the modular and hierarchical nature of plant stress responses and offer potential molecular targets for developing soybean cultivars with enhanced resilience to multiple environmental stresses.

## Materials and methods

### Experimental design and data generation for integrative analysis

The transcriptomic and metabolomic datasets analyzed in this study were generated previously as part of a single comprehensive experiment and are re-analyzed here using an integrative multi-omics framework. The detailed experimental procedures and data generation methods have been described in Castro-Moretti et al. [33] and Husein et al. [34]. Transcriptomic and metabolomic profiles were obtained from adjacent developmental leaves collected from the same plants at identical time points. Briefly, the experiment followed a fully randomized factorial design with three biological replicates per treatment, using the susceptible soybean cultivar BMX Lança IPRO. Plants were grown in a greenhouse until the V4 developmental stage under two water availability regimes: a moderate water deficit (65% of the plant-available soil water) and a well-watered control (80%). Water treatments were initiated 48 h prior to fungal inoculation. Urediniospores of *Phakopsora pachyrhizi* (10^5^ spores mL⁻¹) were sprayed onto the foliage while mock plants received sterile surfactant. Only leaf material collected at 12 and 24 h after inoculation (HAI) from each treatment combination (water status × inoculation), which represent the time points common to both the metabolomic and transcriptomic datasets, were included in the present integrative analysis.

Untargeted metabolomic profiling was performed on V4 leaves using liquid chromatography coupled to mass spectrometry (LC–MS), as detailed by Castro-Moretti et al. [33], enabling the simultaneous detection of primary and secondary metabolites. Transcriptomic data were generated from V3 leaves and processed as described in Husein et al. [34]. RNA-seq reads were aligned to the *Glycine max* Wm82.a4.v1 reference genome using HISAT2, and transcript assembly and quantification were performed with StringTie. Gene-level raw counts were summarized across samples and imported into DESeq2 for normalization prior to WGCNA.

To evaluate complementary aspects of the molecular responses, this study employed two distinct analytical frameworks for multi-omics integration. A linear correlation-based approach and a conditional dependency-based approach were applied independently. These were not applied as a sequential pipeline but as separate frameworks designed to capture distinct association structures. The correlation-based approach was used to identify modules of co-expressed genes and their correlations with metabolites, whereas the conditional dependency approach was conducted independently to infer conditional dependencies among genes, metabolites, and treatment factors.

### Inference of gene–metabolite associations via linear correlation

Raw gene counts were normalized for library size using the median-of-ratios method implemented in DESeq2 [35], which adjusts for sequencing depth and composition bias while maintaining proportional expression patterns across samples. After normalization, genes with low expression (minimum count ≥ 1 in at least 25% of samples) and low variance (retaining the 75% most variable genes) were removed. Outlier samples were identified by hierarchical clustering (hclust, method = “average”) based on sample-to-sample distances, resulting in the exclusion of two samples and a final dataset of 22 samples used for WGCNA construction.

The WGCNA R package was used to construct a signed hybrid co-expression network and to calculate module eigengenes (MEs). Parameters included: soft-thresholding power (β) = 8, selected based on scale-free topology fitting; maxBlockSize = 44,000; networkType = ‘signed hybrid’; correlation type = ‘pearson’; and mergeCutHeight = 0.15, empirically chosen to yield a biologically interpretable number of modules, merging eigengenes with pairwise correlation above 0.85. Hub genes were identified using module membership (MM), representing the Pearson correlation between a gene’s expression and the corresponding ME.

To evaluate the robustness of the identified modules against sample-specific variance, a hub-persistence analysis was performed. This involved 50 bootstrap iterations where the network construction process was repeated on datasets resampled with replacement. For each significant module, the persistence of its biological core was assessed by tracking the co-clustering frequency of its top 10 hub genes, identified via MM. This approach ensures that the reported regulatory anchors are independent of the arbitrary module labeling that occurs during the WGCNA automated merging process.

Linear correlation analysis (Pearson correlation) was performed between MEs and the abundance profiles of the annotated metabolites using the corPvalueStudent function from the WGCNA package. Significant correlations were determined using a strict threshold (*p* < 0.0001), chosen to ensure conservative selection under high-dimensional correlation testing. Significant metabolites were then subjected to hierarchical clustering (hclust, method = ‘average’) applied to the transposed matrix of significant ME-metabolite correlations, using a distance cut-off (h = 1.5) to group metabolites based on their correlation patterns across modules. This threshold was chosen empirically to yield a manageable number of biologically interpretable clusters for downstream analysis.

### Inference of gene–metabolite associations via copula graphical models

To explore conditional dependencies, a Copula Graphical Models (CGM) approach was applied using the nutriNetwork R package. The package is based on the copula graphical model in Behrouzi and Wit [28]. Given the high computational demand of estimating high-dimensional precision matrices, we restricted the input gene set to the unique differentially expressed genes (DEGs) identified across any of the three experimental contrasts (Fungi, Water and Interaction) and both time points, using DESeq2 (|log₂FC| > 1, adjusted p-value < 0.05, Benjamini–Hochberg correction). The same pre-processed expression matrix used in the linear correlation analysis (Sect. 2.2), with outlier samples removed and low-expression genes filtered, was used as the basis for DEG selection.

The input dataset included: (i) expression profiles of the 657 DEGs identified, (ii) abundance profiles of the 455 annotated metabolites, and (iii) a binary incidence matrix encoding the experimental treatment conditions (Fungal Inoculation: 0 = No, 1 = Yes; Water Limitation: 0 = No, 1 = Yes; Sampling Time: 0 = 12 HAI, 1 = 24 HAI). This resulted in a 1,115-variable dataset across the 22 samples. All variables (gene expression, metabolite abundance, incidence matrix columns) were standardized to zero mean and unit variance prior to network construction.

The nutriNetwork function was executed using the Gibbs sampling algorithm (method = “gibbs”) with a fixed regularization parameter (rho = 0.3), selected to balance sparsity and biological interpretability. To justify this selection, a sensitivity analysis was performed across a range of rho values (0.1–0.7). The optimal threshold of rho = 0.3 was identified as the ‘elbow’ point on the network sparsity curve, where edge depletion stabilized following the removal of marginal noise and transitive correlations. Other parameters included em.iter = 5 and em.tol = 0.001. This procedure estimates the copula-based precision matrix (inverse covariance matrix), where non-zero off-diagonal elements represent partial correlations (conditional dependencies) between variables after conditioning on all others. Unlike correlation-based approaches, this allows for the identification of direct associations, removing indirect effects mediated by other variables and providing a more accurate representation of the underlying regulatory structure. The resulting model was post-processed using the selectnet function to extract the final adjacency matrix representing the inferred conditional dependence network. Edges directly connecting the ‘Fungal Inoculation’ or ‘Water Limitation’ nodes to genes or metabolites were extracted to identify variables directly associated with each stress condition within the network structure.

Similar to the robustness test performed for the WGCNA, an edge-selection probability analysis based on 50 bootstrap iterations was conducted for the CGM framework to identify high-confidence associations. This procedure involved repeating the network construction on resampled datasets with replacement to calculate the selection frequency of each edge. Associations were considered robust based on their persistence across iterations, ensuring that the identified stress-specific architectures represent consistent biological dependencies rather than artifacts of sample composition or high-dimensional data noise.

### Functional enrichment analysis

KEGG pathway enrichment analysis was performed using the SoybeanGDB enrichment tool (https://venyao.xyz/SoybeanGDB/, accessed March 28, 2025), as described by Li et al. [36]. Annotation of soybean transcription factors (TFs) was obtained from PlantTFDB 4.0 (Glycine max TF list, downloaded from https://planttfdb.gao-lab.org on March 28, [37], following a characterization of the top 10 hub genes per module. Resistance Gene Analog (RGA) classification and enrichment analysis were conducted following the methodology described by Rody et al. [38], calculating PTI and ETI enrichment scores relative to the total number of RGAs per module and in the genome. General gene functional annotation was based on the Wm82.a4.v1 genome annotation from JGI Phytozome portal (Glycine max Wm82.a4.v1, accessed on March 28, 2025) [39].

## Results

The integration of transcriptomic and metabolomic data using two distinct methodologies enabled the identification of stress-responsive gene-metabolite associations in soybean. The first approach, based on linear correlation analysis, identified marginal associations between co-expressed gene modules and metabolites, providing insight into coordinated metabolic and transcriptional regulation. The second approach, employing copula graphical models, detected probabilistic dependencies, capturing non-linear direct relationships between differentially expressed genes (DEGs) and metabolites under rust infection and water-limiting conditions.

### Correlation-based dependency

**Fig. 1:**
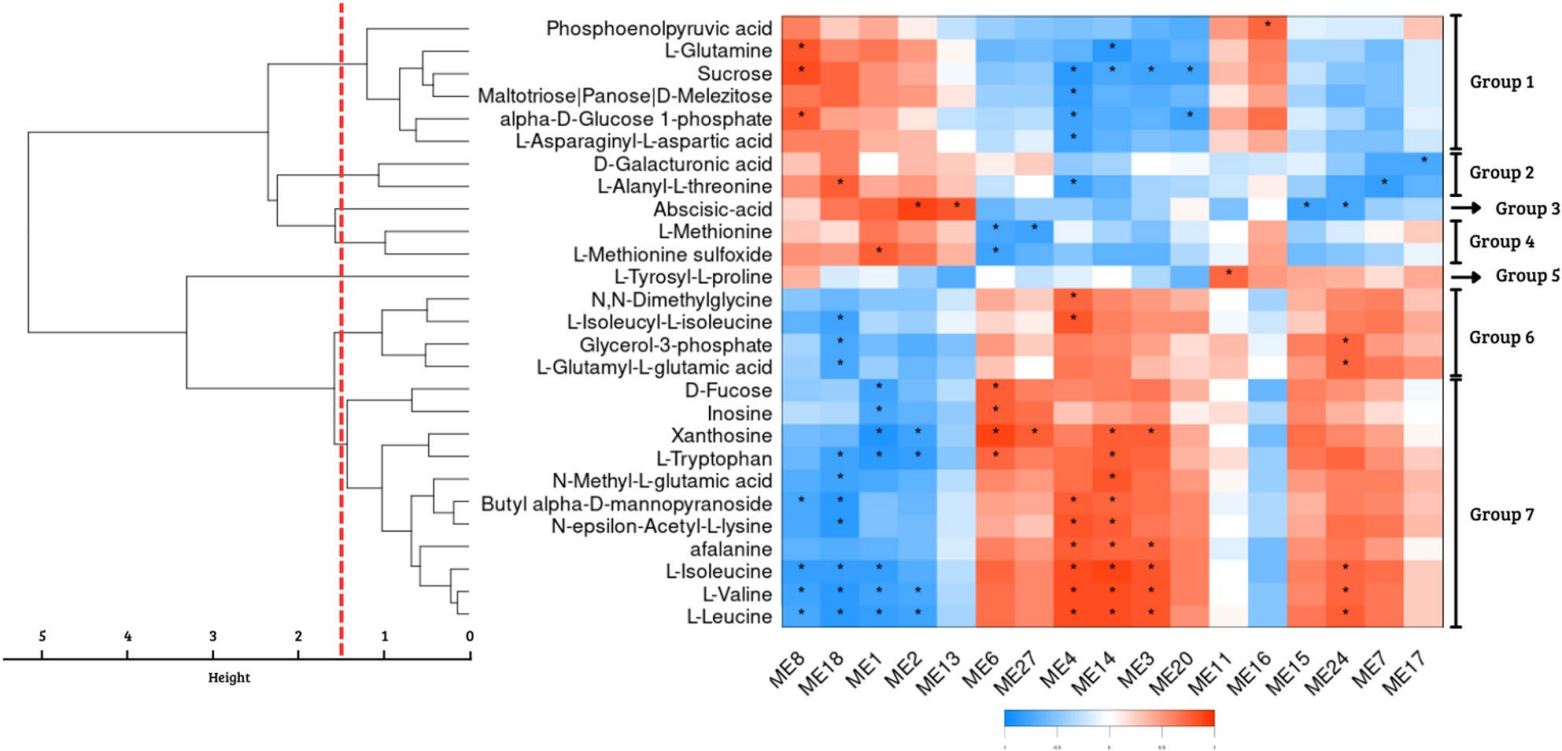
Significant associations between WGCNA modules and metabolites. Significant (*p* < 0.0001) linear association (Pearson correlations) of seventeen WGCNA modules with 27 annotated metabolites. The heatmap displays scaled correlation values between module eigengenes (MEs) and metabolite abundances, with positive correlations shown in red and negative correlations in blue. Asterisks (*) indicate statistically significant associations. Hierarchical clustering of metabolites was performed using complete linkage and Euclidean distance, and the dendrogram was cut at a height of 1.5, defining seven distinct metabolite clusters (Groups 1 to 7), labeled on the right

A co-expression network was constructed from 29,346 genes expressed across two time points and multiple stress conditions using Weighted Gene Co-expression Network Analysis (WGCNA). After merging highly correlated modules (Pearson correlation > 0.85), 32 gene modules were obtained. Pearson correlations were then calculated between these gene modules and the 455 identified metabolites, using a strict significance threshold (*p* < 0.0001). This analysis identified 17 modules with significant associations to 27 metabolites (Fig. 1).

These significant metabolites were clustered into seven groups based on hierarchical clustering with a distance threshold of 1.5 (Fig. 1), Group 1 containing six metabolites (Phosphoenolpyruvic acid, L-glutamine, Sucrose, Maltotriose/Panose/D-melezitose, alpha-D-glucose 1-phosphate and L-asparaginyl-L-aspartic acid), Group 2 with two (D-galacturonic acid and L-alanyl-L-threonine), Group 3 with one (Abscisic acid), Group 4 with two (L-methionine and L-methionine sulfoxide), Group 5 with one (L-tyrosyl-L-proline), Group 6 with four (N, N-dimethylglycine, L-isoleucyl-L-isoleucine, Glycerol-3-phosphate and L-glutamyl-L-glutamic acid) and Group 7 with eleven (D-fucose, Inosine, Xanthosine, L-tryptophan, N-methyl-L-glutamic acid, Butyl alpha-D-mannopyranoside, N-epsilon-acetyl-L-lysine, Afalanine, L-isoleucine, L-valine and L-leucine).

**Fig. 2:**
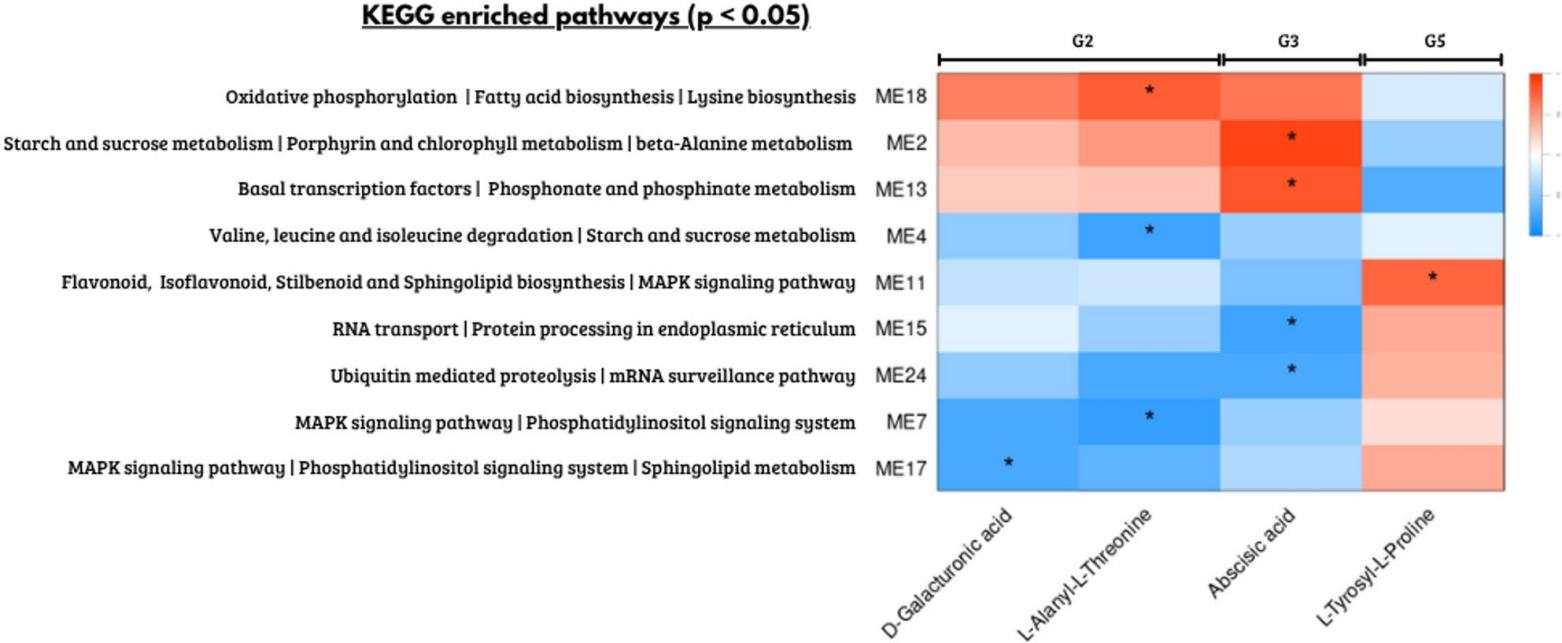
KEGG enriched pathways of gene modules significantly correlated with stress-associated metabolites. Heatmap showing summary KEGG pathways (adjusted *p* < 0.05) enriched in each module with significant linear correlations (Pearson, *p* < 0.0001) between annotated metabolites from Groups 2, 3 and 5. Metabolites were grouped based on correlation patterns, and significant correlations are indicated by asterisks. Red indicates positive correlations and blue negative correlations

From the identified metabolite clusters, three emerged as particularly relevant based on their linear associations with transcriptomic modules (Fig. 2): group 3 was classified as responses to water limitation (abiotic stress), whereas groups 2 and 5 were associated with fungal infection (biotic stress) response.

The hub-persistence analysis revealed high topological stability for the identified stress-responsive modules. The primary modules associated with Abscisic acid (ABA) showed high persistence of their regulatory anchors across 50 bootstrap iterations, with scores of 0.942 (ME2), 0.74 (ME13), 0.728 (ME15), and 0.634 (ME24). Likewise, high stability was observed for modules linked to fungal infection, comprising ME11 (0.782), ME4 (0.96), ME7 (0.908), ME17 (0.86), and ME18 (0.754) (Supplementary Table 1). These results confirm that the reported gene-metabolite associations are anchored by highly stable co-expression structures that are resilient to sample variation.

To further explore the biological roles of the modules associated with stress-responsive metabolites, each significant WGCNA module was functionally characterized through KEGG pathway enrichment, identification of transcription factor (TF) families among the top hub genes, and prediction and enrichment of resistance gene analogs (RGAs). We then assessed their enrichment in RGAs and characterized the TF families represented among their hub genes (Table 1, Supplementary Table 2).

**Table 1:** Functional characterization of gene co-expression modules significantly associated with stress-responsive metabolites

Modules	TF Class	PTI enrichment	ETI enrichment
ME18	—	5.10	7.00
ME2	—	—	—
ME13	DOF, BHLH	—	—
ME4	FAR1	—	—
ME11	—	—	—
ME15	—	3.53	4.96
ME24	GATA	1.76	—
ME7	WRKY	—	—
ME17	NAC, MYB, CAMTA	—	—

Resistance gene analog (RGA) enrichment scores for selected modules were calculated as fold enrichment of genes involved in pattern-triggered immunity (PTI) or effector-triggered immunity (ETI), based on their relative frequency compared to genome-wide RGA distribution. Transcription factor (TF) families were identified among the top 10 hub genes within each module using known TF class annotations. A dash (—) indicates the absence of TFs or no significant RGA enrichment.

**Fig. 3:**
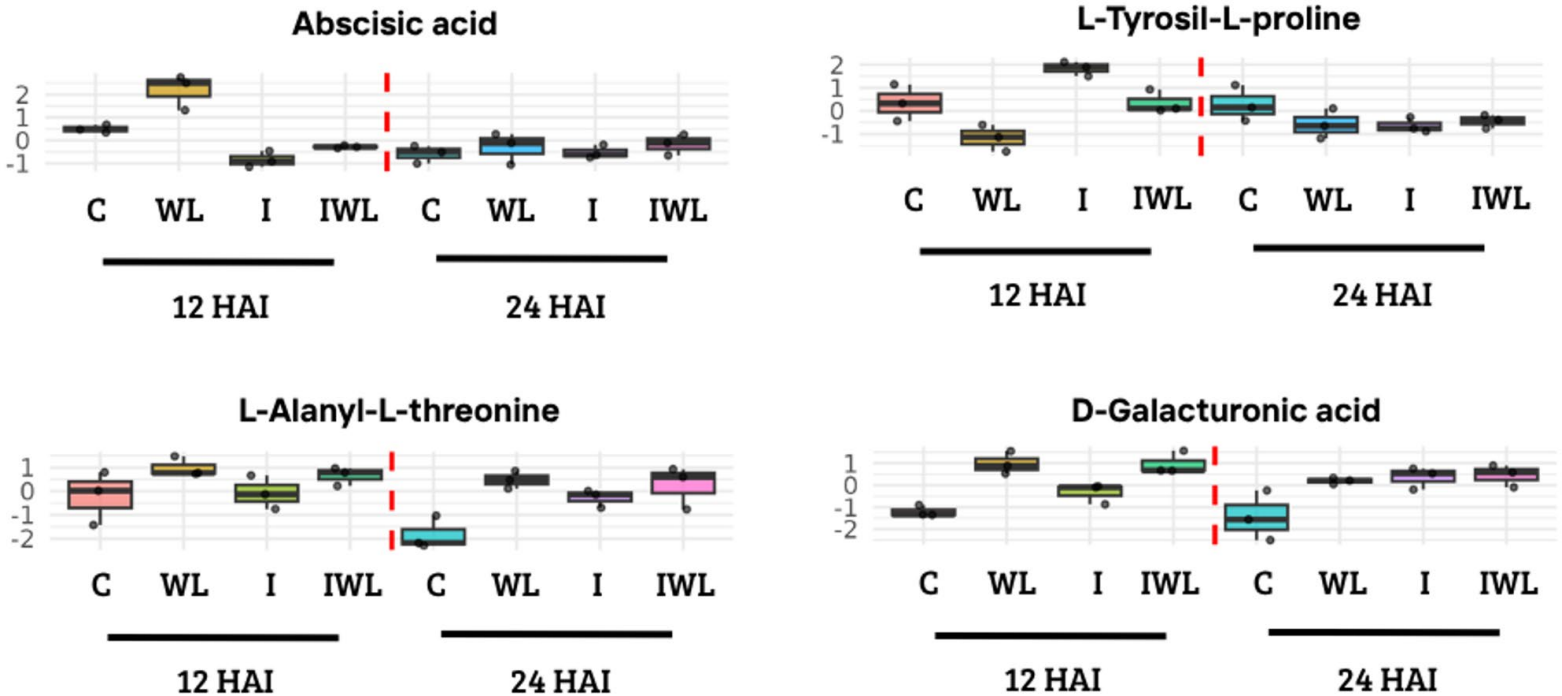
Abundance profiles of selected metabolites across treatments and time points. Boxplots showing the normalized abundance of four metabolites (Abscisic acid, L-tyrosyl-L-proline, L-alanyl-L-threonine, and D-galacturonic acid) in soybean leaves under different treatments: control (C), water limitation (WL), fungal infection (I), and combined stress (IWL), evaluated at 12 and 24 h after inoculation (HAI). Colors represent treatment groups, and each dot corresponds to an individual biological replicate. Each treatment includes three biological replicates (*n* = 3). The boxplots are descriptive representations of metabolite variation across treatments; statistical significance of gene–metabolite associations was assessed separately using Pearson correlation tests with p-values calculated by the corPvalueStudent function in WGCNA. Metabolites were selected based on their stress-specific associations and correlations with gene co-expression modules

Group 3, represented by Abscisic acid, showed a significant linear association with four gene co-expression modules. Modules 2 (*r* = 0.893) and 13 (*r* = 0.805), containing 2,556 and 642 genes respectively, were positively correlated. Their KEGG enrichment (Supplementary Table 3) included pathways such as starch/sucrose metabolism, porphyrin and chlorophyll biosynthesis. Conversely, modules 15 (*r* = -0.767) and 24 (*r* = -0.734), comprising 539 and 207 genes, showed negative correlations and were enriched in processes such as RNA transport, protein processing and ubiquitin-mediated proteolysis. These modules were also enriched for resistance gene analogs (RGAs) related to pattern-triggered immunity (PTI) and effector-triggered immunity (ETI). In terms of transcription factors, DNA binding with one finger (DOF) and basic helix-loop-helix (BHLH) families were enriched in the positively associated modules, while the GATA family was predominant among the negatively associated ones. At the metabolite level, ABA showed increased abundance under water-limiting conditions and reduced abundance in inoculated plants, when compared with the control treatment, exclusively at 12 HAI, with no evident differences between treatments at 24 HAI (Fig. 3).

Group 5, composed of the metabolite L-tyrosyl-L-proline, was only significantly positively correlated with module 11 (*r* = 0.754), which contains 2,235 genes. This module was enriched in KEGG pathways (Supplementary Table 3) related to flavonoid, isoflavonoid, and stilbenoid biosynthesis, as well as sphingolipid metabolism and MAPK signaling pathway, while no enrichment of RGAs or transcription factor families was observed. Consistent with this association, L-tyrosyl-L-proline showed higher abundance in inoculated plants at 12 h after inoculation (HAI) and reduced abundance under water-limiting conditions. At 24 HAI, no clear differences in abundance were observed between treatments (Fig. 3).

Finally, D-galacturonic acid and L-alanyl-L-threonine, components of Group 2, were positively correlated with module 18 (*r* = 0.604; *r* = 0.774), containing 407 genes, and negatively correlated with modules 4 (*r* = -0.463; *r* = -0.766; 2,235 genes), 7 (*r* = -0.721; *r* = -0.827; 1,381 genes) and 17 (*r* = -0.755; *r* = -0.647; 434 genes). Positively correlated KEGG pathway enrichment (Supplementary Table 3) included oxidative phosphorylation, fatty acid biosynthesis, and lysine biosynthesis, while negatively correlated modules were enriched in pathways associated with branched-chain amino acid degradation, autophagy, starch and sucrose metabolism, and MAPK signaling. The positively associated module also showed significant enrichment for RGAs involved in PTI and ETI responses, while no RGA enrichment was found for negatively correlated modules. Among transcription factors, only negatively associated modules were enriched in TF of families FAR1, WRKY, CAMTA, NAC, and MYB. At the metabolite level, L-alanyl-L-threonine exhibited increased abundance in both fungal and water-limited treatments at 24 HAI, with no marked differences at 12 HAI. D-Galacturonic acid showed consistently higher abundance under both biotic and abiotic stress conditions at both 12 and 24 HAI (Fig. 3).

Because eigengene–metabolite correlations were computed across all treatments and time points, significant associations inherently reflect temporal variation in eigengene expression consistent with the metabolite response patterns illustrated in Fig. 3.

### Copula graphical models

A second analytical strategy was employed to detect non-linear and conditional dependencies between genes and metabolites using a copula graphical model. Due to the computational intensity of this method, gene expression data were first filtered to include only differentially expressed genes (DEGs).

**Fig. 4:**
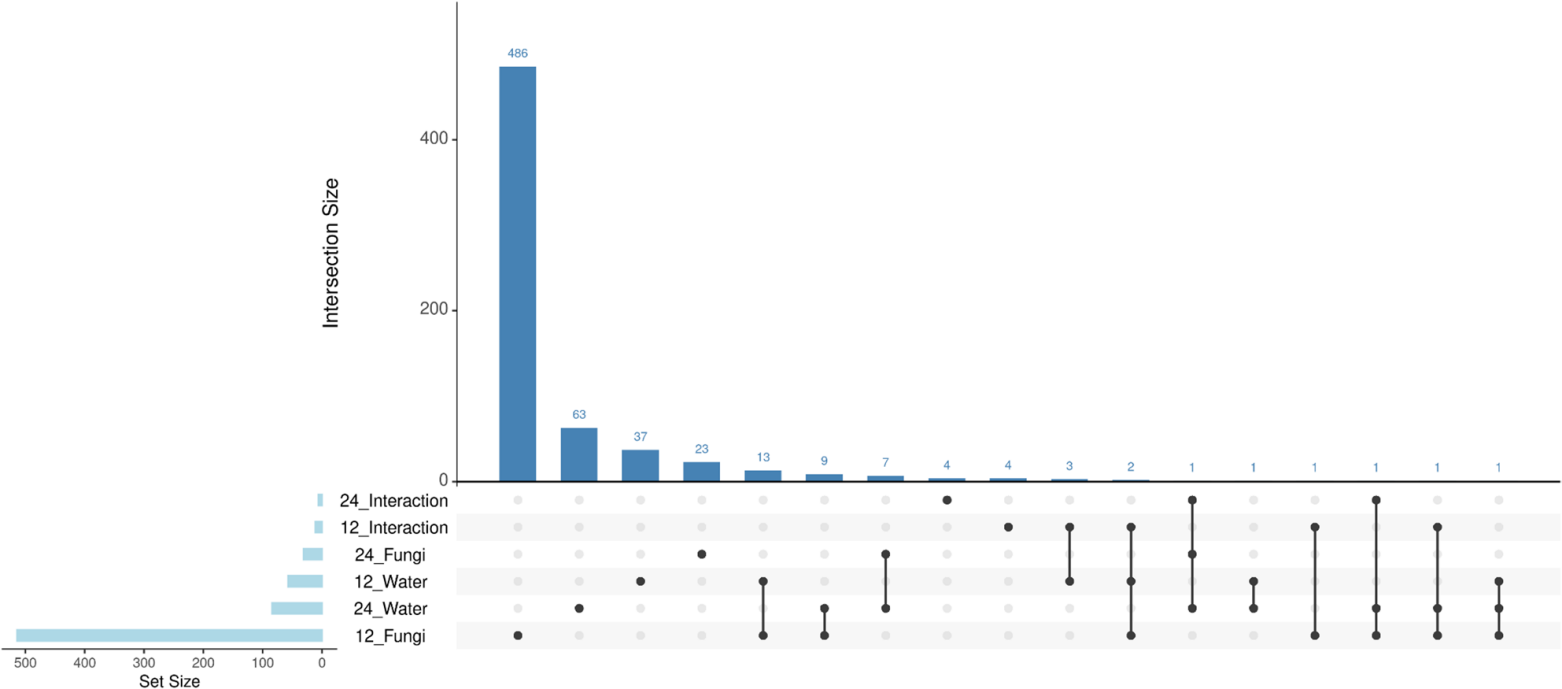
Intersection of differentially expressed genes (DEGs) across stress contrasts and time points. UpSet plot showing the number of DEGs identified under each condition, defined by three contrasts (Fungi infection, Water limitation, Interaction of both stresses) and two time points (12 and 24 HAI). Each horizontal bar on the left represents the total number of DEGs in each condition. The matrix below the vertical bars indicates the combinations of conditions, where shared filled circles denote the presence of an intersection. Vertical bars represent the number of DEGs shared across the respective combination of conditions

A total of 703 DEGs (|log₂FoldChange| > 1, padj < 0.05) were identified across the two collection time points (12 and 24 HAI) and three experimental contrasts: fungal infection by *Phakopsora pachyrhizi*, water limitation, and the interaction of both stresses. Most DEGs were condition-specific, while a subset was shared across two or more contrasts and/or time points (Fig. 4). Among these, 545 DEGs were associated with the biotic (Inoculated vs. non-inoculated), 141 with abiotic stress (water-limited vs. control), and 17 with the interaction between the two stress types. When stratified by sampling time, the majority of DEGs were observed at 12 HAI (582 genes, 82.78%), with a smaller subset detected at 24 HAI (121 genes, 17.21%) (Supplementary Table 4).

**Fig. 5:**
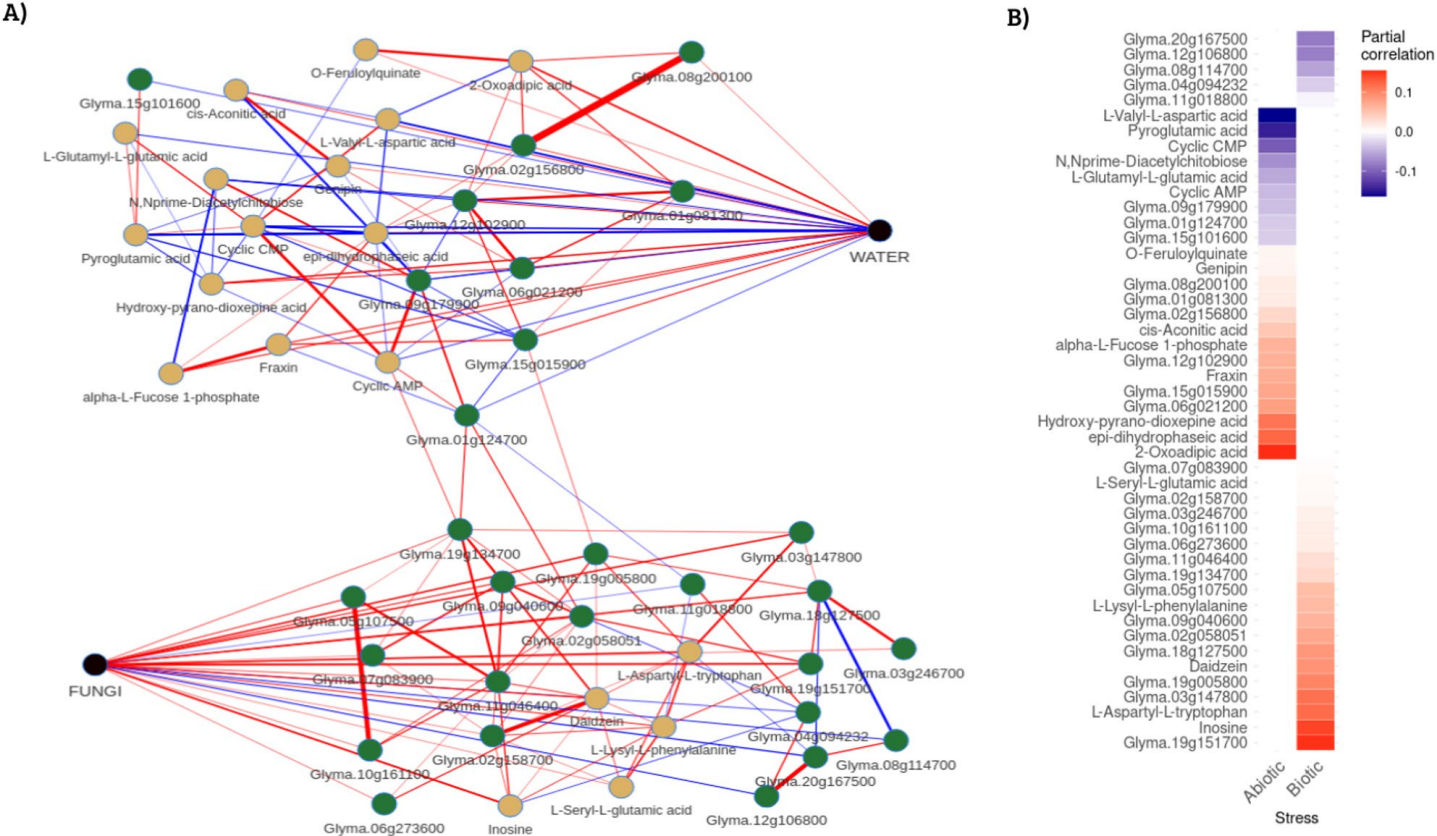
Conditional dependence network inferred by copula graphical modeling. (**A**) Subnetwork extracted from the full copula graphical model showing only the variables directly connected to the biotic (FUNGI) or abiotic (WATER) stress nodes. Nodes represent genes (green) and metabolites (yellow), color-coded according to the direction of their partial correlation with the respective stress condition (blue: negative; red: positive). The central nodes labeled FUNGI and WATER correspond to the binary incidence variables included in the model. Only direct (first-order) associations are displayed. (**B**) Heatmap displaying the magnitude and direction of partial correlations (ρ) for each gene and metabolite directly connected to either the Abiotic (left) or Biotic (right) condition. Positive correlations are shown in red, and negative correlations in blue. Variables are grouped by stress condition, with genes and metabolites ordered by correlation strength within each group

The 657 unique DEGs were integrated with the 455 annotated metabolites and the binary incidence matrix (indicating water status, fungal inoculation, and time) to construct a 1115-variable dataset. The copula graphical model, applied to this matrix, identified partial correlations among variables, revealing conditional dependency structures beyond simple linear associations. Variables were filtered to retain only those directly correlated with the biotic (FUNGI) and abiotic (WATER) stress conditions (Fig. 5A, Supplementary Table 5). The robustness of the inferred stress-specific architectures was further validated through sensitivity and stability analyses. The selection of the regularization parameter rho = 0.3 was justified by a sensitivity analysis evaluating a range of values from 0.1 to 0.7 (Supplementary Fig. 2). A sharp decrease in the number of edges was observed between rho = 0.1 (23,881 edges) and rho = 0.2 (17,351 edges), indicating the successful filtering of likely transitive or indirect correlations. The rate of edge depletion stabilized at rho = 0.3 (14,938 edges), identifying an ‘elbow’ point that maintains a sparse yet biologically informative network structure.

From the copula-based network, a total of 19 genes, 14 with positive and 5 with negative partial correlations, and 5 positively associated metabolites were identified as being connected to fungal stress (Supplementary Table 5). For the water deficit condition, 9 genes (6 positive, 3 negative) and 14 metabolites (8 positive, 6 negative) were identified (Supplementary Table 5). Across both stress conditions, partial correlation coefficients ranged from 0.155 to − 0.164 (Fig. 5B, Supplementary Table 5). Notably, no gene or metabolite was found to be directly associated with both stresses.

For fungal infection, the highest positive correlations were found for the genes *Glyma.19g151700* (ρ = 0.154, Pentatricopeptide repeat superfamily protein) and *Glyma.03g147800* (ρ = 0.117, Disease resistance-responsive (dirigent-like protein) family protein), and for the metabolite Inosine (ρ = 0.145). The most negative correlation was observed with the gene *Glyma.20g167500* (ρ = − 0.084, Abscisic stress ripening-like protein). In response to water stress, the highest positive correlations were observed for the metabolite 2-Oxoadipic acid (ρ = 0.155), Epi-dihydrophaseic acid (ρ = 0.124), Hydroxy-pyrano-dioxepine acid (ρ = 0.114) and the gene *Glyma.06g021200* (ρ = 0.081, Probable nucleoredoxin 2-like isoform 2). The most negative partial correlations were observed for L-Valyl-L-aspartic acid (ρ = − 0.164), Pyroglutamic acid (ρ = − 0.147), and for the gene *Glyma.09g179900* (ρ = − 0.040, Receptor-like kinase).

Complementing this, the edge-selection probability analysis based on 50 bootstrap iterations confirmed the high stability of the primary stress-responsive associations. Among the highest partial correlations with the water limitation factor, the metabolites 2-oxoadipic acid (ρ=0.155) and pyroglutamic acid (ρ =−0.147) exhibited exceptional selection probabilities of 0.94. Additionally, epi-dihydrophaseic acid (ρ=0.124) also showed a probability of 0.94, and L-valyl-L-aspartic acid (ρ =−0.164) presented a probability of 0.90 (Supplementary Table 6). Regarding the associations with the fungal infection factor, high statistical confidence was observed for the top positive association, Glyma.19g151700 (ρ=0.154; 0.74), and for the metabolites L-aspartyl-L-tryptophan (ρ=0.120; 0.80) and inosine (ρ=0.145; 0.64). The gene Glyma.12g106800 (ρ =−0.081), which showed one of the highest negative correlations with the fungus, also exhibited a robust selection probability of 0.68. These results verify that the core architectures defining the stress-specific responses are statistically robust and represent high-confidence molecular signatures of the early soybean defense response.

Although each integrative strategy identified largely distinct gene–metabolite relationships, a few overlapping features emerged, reinforcing the biological consistency of the integrative findings. The gene *Glyma.08g114700* (calmodulin-binding protein) exhibited a negative partial correlation with the presence of the fungus in the CGM (ρ = − 0.058), and was also a member of WGCNA Module 17, which was negatively correlated with Group 2 (metabolite cluster enriched in biotic response signatures). Similarly, *Glyma.06g021200* (probable nucleoredoxin), *Glyma.12g102900* (beta-amylase 5), *Glyma.01g081300* (alpha-glucosidase), and *Glyma.08g200100* (HAD superfamily phosphatase) were positively associated with the water-limiting condition in the CGM and simultaneously located in WGCNA modules positively correlated with Group 3, represented by abscisic acid.

## Discussion

The integrative strategy employed in this study, combining transcriptomic and metabolomic data based on linear associations (Pearson correlation) and conditional dependency inference (CGM), revealed discrete regulatory architectures underlying soybean responses to ASR and water limitation. Notably, no gene or metabolite was directly associated with both stress conditions, supporting a model in which soybean activates stress-specific modules rather than shared signaling pathways under concurrent challenges. This separation was consistently observed in both the correlation-based and conditional models, suggesting that each stressor elicits a dedicated molecular response with minimal overlap. It is important to note that the apparent absence of shared features between stresses may also reflect the early sampling window (12 to 24 HAI) and conservative filtering applied to ensure high confidence in associations. Therefore, this result should be interpreted as indicative of predominant modularity rather than complete exclusivity.

Interestingly, the response uncovered by the integrated analysis differed from those observed in the transcriptomic and metabolomic datasets individually: while the transcriptome revealed a unique expression state under stress combination [34], and the metabolome showed time-dependent modulations of specific compounds [33], the current multi-omic approach, in contrast, highlights the preservation of stress-specific responses even under simultaneous exposure, reflecting a modular regulatory strategy likely evolved to mitigate antagonistic crosstalk between signaling pathways such as those mediated by abscisic acid (ABA) versus pathogen-induced responses, allowing the plant to allocate metabolic resources according to the specific stress condition [40]. This pattern suggests that drought- and rust-associated programs operate in parallel during early responses to combined stress, indicating limited antagonism rather than mutual exclusion. Thus, analyzing the combined stress provides insight into how soybean balances potentially conflicting signaling branches within the same physiological context. Although the factorial design included both single- and combined-stress treatments, the small number of interaction-specific DEGs observed further supports this modular behavior revealed by the integrative analysis. The high topological stability observed in the hub-persistence analysis confirms that the modules represent genuine co-expression units, despite the network being constructed using pooled samples from all four treatments. This global approach allowed for the identification of regulatory architectures that remain cohesive across the full factorial experimental design, providing a systemic view of how different stress-responsive branches are activated in parallel. The findings reinforce the need for integrative approaches that distinguish between coexpression and direct interaction, especially when investigating multifactorial stress contexts.

Among all metabolites detected, ABA was the most consistently associated with the water-limiting treatment, identified by both linear correlation and CGM approaches. This finding is in line with the central role of ABA in plant drought responses, as it is widely recognized as the primary phytohormone mediating adaptive responses to water deficit through rapid accumulation, stomatal closure, and transcriptional reprogramming, resulting in the expression of drought-responsive genes [41, 42]. In the correlation-based analysis, ABA was positively correlated with modules enriched in primary metabolic pathways, such as starch and sucrose metabolism, which are commonly affected under drought stress [43]. The presence of Dof and bHLH transcription factors (TF) in these modules suggests regulatory coordination of metabolic and developmental pathways under ABA influence. Prior studies have demonstrated that these TF families promote drought tolerance in tomato and peanut, primarily by enhancing ABA accumulation, increasing ROS scavenging, and repressing ABA catabolic activity [44, 45]. In contrast, modules showing negative correlations with ABA were associated with RNA and protein-related processes, including ubiquitin-dependent protein degradation. This negative association likely reflects a shift in regulatory priorities under drought-induced ABA signaling. As reviewed by Xu et al. (2025) [46], elevated ABA levels modulate the ubiquitin–proteasome system (UPS) to promote the selective degradation of transcriptional regulators and signaling components, prioritizing stress adaptation over growth-related processes. These modules also exhibited enrichment for resistance gene analogs (RGAs) from both PTI and ETI classes. Although RGAs are typically implicated in biotic defense, their repression in response to water deficit suggests an antagonistic regulation between abiotic and biotic responses, possibly mediated by ABA-induced suppression of defense pathways. Supporting this view, ABA was shown to promote immune suppression in *Arabidopsis* through the induction of HAI-type phosphatases that inactivate immune-associated MAPKs, revealing a mechanistic basis for ABA-mediated antagonism of immune pathways [47].

The CGM analysis yielded a narrower and more stringent subset of directly associated features, identifying nine genes and fourteen metabolites with direct conditional dependencies on the water stress factor. Among these, Glyma.06g021200, encoding a nucleoredoxin, was directly linked to the water condition node and may act as a key modulator of redox homeostasis under water limitation. Nucleoredoxins are oxidoreductases known to preserve the activity of antioxidant enzymes, such as catalase under ROS-enriched conditions. In Arabidopsis, the activity of catalase under oxidative conditions is supported by NRX1, which helps preserve its functionality during oxidative stress [48]. In wheat, overexpression of TaNRX1 enhances drought tolerance by boosting osmoprotectant accumulation, increasing antioxidant enzyme activity, and modulating stress-related gene expression [49]. These observations align with the hypothesis that Glyma.06g021200 may act independently of ABA signaling to maintain redox balance under water limitation conditions. Additionally, the presence of metabolites such as 2-oxoadipic acid and epi-dihydrophaseic acid (epi-DPA) suggests targeted metabolic regulation under water limitation conditions. The former, involved in lysine and tryptophan degradation, was also found in roots of drought-stressed pine seedlings, implying a contribution to amino acid turnover and redox buffering [50]. The latter is a byproduct of ABA catabolism, accumulated during ABA deactivation, marking the cessation of ABA signaling and the transition to homeostatic recovery [51]. Together, the presence of redox-related genes and hormone-catabolizing metabolites is consistent with a coordinated stress response involving both ABA deactivation and metabolic adjustment to restore redox equilibrium.

In contrast to the metabolite-dominated response observed under water limitation, the response to *P. pachyrhizi* infection was dominated by transcriptional reprogramming. The CGM analysis identified nineteen genes and five metabolites conditionally dependent on the biotic treatment, including genes encoding a disease resistance-responsive (dirigent-like protein) family and pentatricopeptide repeat (PPR) proteins. Dirigent-like proteins, such as CaDIR7 in *Capsicum annuum*, are involved in lignin biosynthesis and defense against *Phytophthora spp.*, reinforcing cell walls to hinder pathogen invasion [52]. Similarly, PPR proteins mediate organellar gene expression by controlling RNA metabolism, and several have been implicated in defense signaling and stress adaptation via chloroplast and mitochondrial regulation [53]. The limited number of associated metabolites suggests that the early stages of the response toP. pachyrhizi for this susceptible cultivar favor signal perception and transcriptional reprogramming over broad metabolic shifts, a pattern consistent with rapid activation of immune regulatory cascades [54]. Interestingly, modules negatively correlated with biotic stress-related metabolites identified using the correlation-based approach were enriched for WRKY, NAC, MYB, and CAMTA transcription factors, which are key regulators of biotic and abiotic stress response [55, 56], but lacked enrichment for classical RGAs. This configuration likely reflects pathogen-mediated suppression of host signaling, which has been explicitly described in the *Glycine max–Phakopsora pachyrhizi* pathosystem, where secreted effectors suppress basal defenses to enable effector-triggered susceptibility (ETS) in compatible interactions [57, 58]. The lack of RGA enrichment during these early stages, together with the expression of stress-related transcription factors, is consistent with observations in other susceptible soybean genotypes, where a limited or incomplete immune activation has been associated with reduced NLR expression, suggesting that attenuated immune signaling contributes to disease susceptibility [59].

Among the metabolites associated with the rust presence, inosine exhibited the strongest positive partial correlation with infection, suggesting a potential role in early immune regulation. In plants, the accumulation of inosine di- and triphosphate, resulting from a deficiency in inosine triphosphate pyrophosphatase (ITPA), leads to the accumulation of salicylic acid, an immune response, and premature senescence, indication a signaling function in stress perception conserved in many organisms [60]. Moreover, in animal models, inosine has been shown to fine-tune innate immunity by suppressing pro-inflammatory cytokines such as IL-1β while enhancing complement system activation, thus coordinating cellular and humoral immune responses during bacterial infection [61]. Although the functional role of inosine in soybean immunity remains to be elucidated, its accumulation under fungal infection may reflect a conserved metabolic strategy across kingdoms to modulate biotic defense responses.

In the correlation-based integrative analysis, three metabolites emerged as particularly relevant for *P. pachyrhizi* infection, combining statistical robustness with biological relevance. Two of them, L-alanyl-L-threonine and L-tyrosyl-L-proline, suggest functional links to amino acid-mediated defense mechanisms. The former, though not directly linked to plant immunity, may accumulate as part of the host’s early biochemical response to fungal invasion, given the role of dipeptides in defense metabolite biosynthesis and immune signaling [62]. L-tyrosyl-L-proline combines two stress-related amino acids: tyrosine, which is central to antioxidant systems and the production of lignin and phytoalexins [63], and proline, known for its osmoprotective and redox-balancing effects under stress [64]. Although the functional role of L-tyrosyl-L-proline in soybean immunity is uncharacterized, its positive correlation with a module enriched in MAPK signaling and flavonoid-related biosynthesis supports its possible involvement in defense regulation. D-Galacturonic acid, also highlighted, is a component of oligogalacturonides, which act as elicitors of immune responses [65, 66]. Though less active than the full oligomer, its accumulation may reflect pectin degradation initiated during fungal entry. This is consistent with the known infection dynamics of *P. pachyrhizi*, which establishes haustoria within the first 24 h [58]. Together, these metabolites may represent distinct biochemical facets of the biotic stress response, potentially linking early signaling, metabolic adjustment, and structural remodeling in the absence of classical immune activation.

The use of both WGCNA and CGM enabled a multi-layered interpretation of gene–metabolite associations by distinguishing broad coexpression patterns from direct conditional dependencies. WGCNA revealed coordinated transcriptional modules associated with stress-induced metabolic changes, providing an overview of systemic regulatory responses. However, such correlation-based analyses capture both direct and indirect relationships, potentially obscuring key regulatory nodes. In contrast, CGM estimated conditional dependencies among variables, identifying sparse, high-confidence networks in which only direct interactions are retained. Importantly, even near-zero partial correlations may capture meaningful associations in the copula-transformed space, since these values are calculated after correcting for all other variables in the dataset [32, 67, 68]. The non-overlapping sets of genes and metabolites identified by each approach emphasize their analytical complementarity, as they capture different aspects of molecular regulation, and their integration offers a more accurate prioritization of molecular targets for functional studies under complex stress conditions. This analytical complementarity, even when applied independently, allows CGM to refine and prioritize associations detected by WGCNA rather than duplicate them. Future studies may benefit from adopting this dual-framework strategy as a standard in multi-omics integration, particularly when aiming to disentangle context-specific regulatory circuits in plant stress biology. Given the modest sample size (*n* = 22), the networks inferred here should be interpreted as exploratory but biologically guided representations. The consistency of stress-specific associations across both WGCNA and CGM, despite their different statistical frameworks, supports the robustness of the main patterns observed.

The stress-specific molecular signatures identified in this study provide a valuable resource for breeding programs aiming to enhance tolerance to abiotic and biotic stresses simultaneously. The absence of shared genes or metabolites between stress conditions implies that dual stress tolerance cannot be efficiently obtained by selecting for markers that are simultaneously associated with both stresses. Instead, targeted stacking of stress-specific regulators, carefully chosen to avoid antagonistic cross-regulation, emerges as a more promising strategy. Among the conditionally associated genes identified by CGM, *Glyma.06g021200* (nucleoredoxin), *Glyma.19g151700* (PPR protein) and *Glyma.03g147800* (dirigent-like protein) represent particularly compelling candidates for functional characterization. These genes are directly associated with water limitation (first gene) and rust infection (last two genes) responses, and their respective roles in redox homeostasis, RNA metabolism, signaling, and pathogen response, position them as putative hubs for engineering enhanced stress resilience. Similarly, metabolites such as ABA, 2-oxoadipic acid, and inosine, which exhibited stress-specific associations, may serve as reliable biochemical markers for phenotyping early-stage responses in field conditions. The observed conditional independence between responses also suggests that improvements in one trait may not incur penalties in the other, provided that antagonistic regulators like ABA are carefully managed. These insights offer a rational framework for designing molecular breeding strategies that maximize multi-stress resilience while minimizing trade-offs.

However, the use of only the susceptible cultivar inherently limits the generality of our conclusions. Future experiments that include resistant genotypes will be essential to disentangle susceptibility-specific effects from universal stress responses. Another consideration is that the transcriptomic and metabolomic profiles were obtained from adjacent developmental leaves (V3 and V4) collected from the same plants and at identical time points. This sampling strategy minimized environmental variation while ensuring that both omic layers captured coordinated physiological responses. Nevertheless, slight developmental differences between adjacent leaves may have influenced the strength of specific gene–metabolite correlations, and this limitation should be considered when interpreting direct cross-omic relationships. Moreover, our focus on early stages (12 and 24 HAI) and the use of DEG-based pre-filtering captures the primary signaling phase and high-confidence molecular blueprints, it may have overlooked later-emerging or shared interactions that appear as stress responses become generalized. Thus, extending sampling to later stages or broader gene sets could uncover additional layers of drought–pathogen interplay. Lastly, the functional roles proposed for specific genes and metabolites, while these interpretations are supported by published evidence in soybean and other species, they remain working hypotheses that require experimental validation in soybean. The associations identified here should therefore be viewed as biologically grounded leads for future functional studies. Future analyses incorporating promoter motif enrichment or TF binding-site prediction could help verify the regulatory influence of the transcription factor hubs identified here and strengthen the mechanistic interpretation of the inferred co-expression networks.

By integrating transcriptomic and metabolomic data through both correlation-based and conditional dependency frameworks, this study delineates distinct regulatory architectures underlying soybean responses to water limitation and rust infection. The identification of stress-specific modules, the central role of ABA in drought adaptation, and the transcriptional reprogramming observed during pathogen challenge highlight the modular and hierarchical nature of these responses. The complementarity between WGCNA and CGM proved essential for refining candidate genes and metabolites, offering a robust platform for future functional validation. These findings advance our understanding of how soybean orchestrates specialized responses to complex environmental stimuli and provide actionable molecular targets for developing cultivars with improved resilience to concurrent abiotic and biotic stress. These patterns support modular and parallel responses with limited antagonism between stress pathways, suggesting that resilience can be enhanced by stacking condition-specific regulators while minimizing potential cross-talk.

## Supplementary Information

Supplementary Material 1.

Supplementary Material 2.

Supplementary Material 3.

Supplementary Material 4.

Supplementary Material 5.

Supplementary Material 6.

Supplementary Material 7.

Supplementary Material 8.

Supplementary Material 9.

## Data Availability

All scripts used in this study are available at the GitHub repository (https://github.com/GustavoHusein/Soybean-integration-Analysis/tree/main/Soybean-integration-Analysis). Sequence data from this study have been submitted to the NCBI Sequence Read Archive under accession PRJNA1137319.

## References

[1] Rahman SU, et al. Improvement of Soybean; A Way Forward Transition from Genetic Engineering to New Plant Breeding Technologies. Mol Biotechnol. 2023;65:162–80. 35119645 10.1007/s12033-022-00456-6

[2] Dean R, et al. The Top 10 fungal pathogens in molecular plant pathology. Mol Plant Pathol. 2012;13:804–804. 10.1111/j.1364-3703.2011.00783.x PMC6638784 22471698

[3] Alves MDC, De Carvalho LG, Pozza EA, Sanches L, Maia JC. D. S. Ecological zoning of soybean rust, coffee rust and banana black sigatoka based on Brazilian climate changes. Procedia Environ Sci. 2011;6:35–49.

[4] Burdon JJ, Zhan J. Climate change and disease in plant communities. PLoS Biol. 2020;18:e3000949. 33232314 10.1371/journal.pbio.3000949 PMC7685433

[5] Delgado-Baquerizo M, et al. The proportion of soil-borne pathogens increases with warming at the global scale. Nat Clim Change. 2020;10:550–4.

[6] Ghini R, Hamada E, Gonçalves RRV, Gasparotto L, Pereira JC. R. Análise de risco das mudanças climáticas globais sobre a sigatoka-negra da bananeira no Brasil. Fitopatol Bras. 2007;32:197–204.

[7] Leng G, Hall J. Crop yield sensitivity of global major agricultural countries to droughts and the projected changes in the future. Sci Total Environ. 2019;654:811–21. 30448671 10.1016/j.scitotenv.2018.10.434 PMC6341212

[8] Thornton PK, Ericksen PJ, Herrero M, Challinor AJ. Climate variability and vulnerability to climate change: a review. Glob Change Biol. 2014;20:3313–28. 10.1111/gcb.12581 PMC4258067 24668802

[9] Rosa ED. C. R. Asian Soybean Rust Resistance: An Overview. 2015. J Plant Pathol Microbiol 06.

[10] Camejo D, et al. High temperature effects on photosynthetic activity of two tomato cultivars with different heat susceptibility. J Plant Physiol. 2005;162:281–9. 15832680 10.1016/j.jplph.2004.07.014

[11] Gerós H, Chaves MM, Gil HM, Delrot SA, Molecular and Ecophysiological, Perspective. 2016.

[12] Mittler R. Abiotic stress, the field environment and stress combination. Trends Plant Sci. 2006;11:15–9. 16359910 10.1016/j.tplants.2005.11.002

[13] Trivedi P, Batista BD, Bazany KE, Singh BK. Plant-microbiome interactions under a changing world: responses, consequences and perspectives. New Phytol. 2022;234:1951–9. 35118660 10.1111/nph.18016

[14] Kakumanu A, et al. Effects of Drought on Gene Expression in Maize Reproductive and Leaf Meristem Tissue Revealed by RNA-Seq1[W][OA]. Plant Physiol. 2012;160:846–67. 22837360 10.1104/pp.112.200444 PMC3461560

[15] Le DT, et al. Differential Gene Expression in Soybean Leaf Tissues at Late Developmental Stages under Drought Stress Revealed by Genome-Wide Transcriptome Analysis. PLoS ONE. 2012;7:e49522. 23189148 10.1371/journal.pone.0049522 PMC3505142

[16] Xue Y, et al. Genome-wide association analysis for nine agronomic traits in maize under well-watered and water-stressed conditions. Theor Appl Genet. 2013;126:2587–96. 23884600 10.1007/s00122-013-2158-x

[17] Roychowdhury R, et al. Multi-Omics Pipeline and Omics-Integration Approach to Decipher Plant’s Abiotic Stress Tolerance Responses. Genes. 2023;14:1281. 37372461 10.3390/genes14061281 PMC10298225

[18] Sarfraz Z, et al. Plant Biochemistry in the Era of Omics: Integrated Omics Approaches to Unravel the Genetic Basis of Plant Stress Tolerance. Plant Breed pbr. 2025;13277. 10.1111/pbr.13277.

[19] Eicher T, et al. Metabolomics and Multi-Omics Integration: A Survey of Computational Methods and Resources. Metabolites. 2020;10:202. 32429287 10.3390/metabo10050202 PMC7281435

[20] Langfelder P, Horvath S. WGCNA: an R package for weighted correlation network analysis. BMC Bioinformatics. 2008;9:559. 19114008 10.1186/1471-2105-9-559 PMC2631488

[21] Sanches PHG, de Melo NC, Porcari AM, de Carvalho LM. Integrating Molecular Perspectives: Strategies for Comprehensive Multi-Omics Integrative Data Analysis and Machine Learning Applications in Transcriptomics, Proteomics, and Metabolomics. Biology. 2024;13:848. 39596803 10.3390/biology13110848 PMC11592251

[22] Kao P-H, et al. Identification of key drought-tolerant genes in soybean using an integrative data-driven feature engineering pipeline. J Big Data. 2025;12:68.

[23] Ovens K, Eames BF, McQuillan I. Comparative Analyses of Gene Co-expression Networks: Implementations and Applications in the Study of Evolution. Front Genet. 2021;12:695399. 34484293 10.3389/fgene.2021.695399 PMC8414652

[24] Behrouzi P, et al. Dietary Intakes of Vegetable Protein, Folate,and Vitamins B-6 and B-12 Are Partially Correlated with Physical Functioning of Dutch Older Adults Using Copula Graphical Models. J Nutr. 2020;150:634–43. 31858107 10.1093/jn/nxz269 PMC7056616

[25] Farnoudkia H, Purutcuoglu V. Copula Gaussian graphical modeling of biological networks and Bayesian inference of model parameters. 2019. Scientia Iranica 26.

[26] Dobra A, Lenkoski A. Copula Gaussian graphical models and their application to modeling functional disability data. 2011. Ann Appl Stat 5.

[27] Liu H, Lafferty J, Wasserman L. The Nonparanormal: Semiparametric Estimation of High Dimensional Undirected Graphs 2009.

[28] Behrouzi P, Wit EC. Models J R Stat Soc Ser C Appl Stat. 2019;68:141–60. Detecting Epistatic Selection with Partially Observed Genotype Data by Using Copula Graphical.

[29] Hermes S, Van Heerwaarden J, Behrouzi P. Copula Graphical Models for Heterogeneous Mixed Data. J Comput Graph Stat. 2024;33:991–1005.

[30] Hermes S, Van Heerwaarden J, Behrouzi P. Using copula graphical models to detect the impact of drought stress on maize and wheat yield. Silico Plants. 2023;5:diad008.

[31] Prado M et al. Complementary approaches to dissect late leaf rust resistance in an interspecific raspberry population. 2024. G3 GenesGenomesGenetics 14, jkae202. 10.1093/g3journal/jkae202 PMC11457092 39172650

[32] Grootswagers P, et al. Discovering the direct relations between nutrients and epigenetic ageing. J Nutr Health Aging. 2024;28:100324. 39067141 10.1016/j.jnha.2024.100324 PMC12880071

[33] Castro-Moretti FR et al. Water Limitation Causes Early-Stage Metabolic Perturbation in the Interaction of Soybean and the Causal Agent of Asian Soybean Rust. 2026. J Agric Food Chem. 10.1021/acs.jafc.5c07944 PMC12879939 41574735

[34] Husein G, et al. Transcriptome Profiling of Resistance Genes Analogs in Soybean’s Cross-Tolerance to Water Limitation and Rust Stress. Food Energy Secur. 2025;v14:n5.

[35] Love MI, Huber W, Anders S. Moderated estimation of fold change and dispersion for RNA-seq data with DESeq2. Genome Biol. 2014;15:550. 25516281 10.1186/s13059-014-0550-8 PMC4302049

[36] Li H, et al. SoybeanGDB: A comprehensive genomic and bioinformatic platform for soybean genetics and genomics. Comput Struct Biotechnol J. 2023;21:3327–38. 38213885 10.1016/j.csbj.2023.06.012 PMC10781885

[37] Jin J, et al. PlantTFDB 4.0: toward a central hub for transcription factors and regulatory interactions in plants. Nucleic Acids Res. 2017;45:D1040–5. 27924042 10.1093/nar/gkw982 PMC5210657

[38] Rody HVS, et al. Genome survey of resistance gene analogs in sugarcane: genomic features and differential expression of the innate immune system from a smut-resistant genotype. BMC Genomics. 2019;20:809. 31694536 10.1186/s12864-019-6207-y PMC6836459

[39] Goodstein DM, et al. Phytozome: a comparative platform for green plant genomics. Nucleic Acids Res. 2012;40:D1178–86. 22110026 10.1093/nar/gkr944 PMC3245001

[40] Anderson JP, et al. Antagonistic Interaction between Abscisic Acid and Jasmonate-Ethylene Signaling Pathways Modulates Defense Gene Expression and Disease Resistance in Arabidopsis. Plant Cell. 2004;16:3460–79. 15548743 10.1105/tpc.104.025833 PMC535886

[41] Margay AR, Mehmood A, Bashir L. Review on Hormonal Regulation of Drought Stress Response in Plants. Int J Plant Soil Sci. 2024;36:902–16.

[42] Muhammad Aslam M, et al. Mechanisms of Abscisic Acid-Mediated Drought Stress Responses in Plants. Int J Mol Sci. 2022;23:1084. 35163008 10.3390/ijms23031084 PMC8835272

[43] Nidumolu LCM, Lorilla KM, Chakravarty I, Uhde-Stone C. Soybean Root Transcriptomics: Insights into Sucrose Signaling at the Crossroads of Nutrient Deficiency and Biotic Stress Responses. Plants. 2023;12:2117. 37299096 10.3390/plants12112117 PMC10255639

[44] Liang Y, et al. A bHLH transcription factor, SlbHLH96, promotes drought tolerance in tomato. Hortic Res. 2022;9:uhac198. 36467272 10.1093/hr/uhac198 PMC9714257

[45] Liu Z, et al. Landscape of rare-allele variants in cultivated and wild soybean genomes. Plant Genome. 2025;18:e70020. 40148071 10.1002/tpg2.70020 PMC11949740

[46] Xu J, et al. The ubiquitin-proteasome system in the plant response to abiotic stress: Potential role in crop resilience improvement. Plant Sci. 2024;342:112035. 38367822 10.1016/j.plantsci.2024.112035

[47] Mine A, et al. Pathogen exploitation of an abscisic acid- and jasmonate-inducible MAPK phosphatase and its interception by Arabidopsis immunity. Proc Natl Acad Sci. 2017;114:7456–61. 28652328 10.1073/pnas.1702613114 PMC5514735

[48] Kneeshaw S, et al. Nucleoredoxin guards against oxidative stress by protecting antioxidant enzymes. Proc Natl Acad Sci. 2017;114:8414–9. 28724723 10.1073/pnas.1703344114 PMC5547615

[49] Zhang Y, et al. Nucleoredoxin Gene TaNRX1 Positively Regulates Drought Tolerance in Transgenic Wheat (Triticum aestivum L.). Front. Plant Sci. 2021;12:756338. 10.3389/fpls.2021.756338 PMC8632643 34868149

[50] Wu C, Wang Y, Sun H. Targeted and untargeted metabolomics reveals deep analysis of drought stress responses in needles and roots of Pinus taeda seedlings. Front. Plant Sci. 2023;13:1031466. 10.3389/fpls.2022.1031466 PMC9927248 36798806

[51] Bai Y-L, et al. Neophaseic acid catabolism in the 9′-hydroxylation pathway of abscisic acid in Arabidopsis thaliana. Plant Commun. 2022;3:100340. 35585783 10.1016/j.xplc.2022.100340 PMC9482987

[52] Khan A, et al. Genome-wide analysis of dirigent gene family in pepper (Capsicum annuum L.) and characterization of CaDIR7 in biotic and abiotic stresses. Sci Rep. 2018;8:5500. 29615685 10.1038/s41598-018-23761-0 PMC5883049

[53] Meng L, et al. PPR proteins in plants: roles, mechanisms, and prospects for rice research. Front Plant Sci. 2024;15:1416742. 38993942 10.3389/fpls.2024.1416742 PMC11236678

[54] Meraj TA, et al. Transcriptional Factors Regulate Plant Stress Responses Through Mediating Secondary Metabolism. Genes. 2020;11:346. 32218164 10.3390/genes11040346 PMC7230336

[55] Bian Z, Gao H, Wang CNAC. Transcription Factors as Positive or Negative Regulators during Ongoing Battle between Pathogens and Our Food Crops. Int J Mol Sci. 2020;22:81. 33374758 10.3390/ijms22010081 PMC7795297

[56] Li S, et al. WRKY Transcription Factors (TFs) as Key Regulators of Plant Resilience to Environmental Stresses: Current Perspective. Agronomy. 2024;14:2421.

[57] Chicowski AS, Bredow M, Utiyama AS, Marcelino-Guimarães FC, Whitham SA. Soybean-Phakopsora pachyrhizi interactions: towards the development of next-generation disease-resistant plants. Plant Biotechnol J. 2024;22:296–315. 37883664 10.1111/pbi.14206 PMC10826999

[58] Gupta YK, et al. Major proliferation of transposable elements shaped the genome of the soybean rust pathogen Phakopsora pachyrhizi. Nat Commun. 2023;14:1835. 37005409 10.1038/s41467-023-37551-4 PMC10067951

[59] Hao Q, et al. RNA-Seq and Comparative Transcriptomic Analyses of Asian Soybean Rust Resistant and Susceptible Soybean Genotypes Provide Insights into Identifying Disease Resistance Genes. Int J Mol Sci. 2023;24:13450. 37686258 10.3390/ijms241713450 PMC10487414

[60] Straube H, et al. An inosine triphosphate pyrophosphatase safeguards plant nucleic acids from aberrant purine nucleotides. New Phytol. 2023;237:1759–75. 36464781 10.1111/nph.18656

[61] Jiang M, et al. Succinate and inosine coordinate innate immune response to bacterial infection. PLOS Pathog. 2022;18:e1010796. 36026499 10.1371/journal.ppat.1010796 PMC9455851

[62] Parthasarathy A, Borrego EJ, Savka MA, Dobson RCJ, Hudson AO. Amino acid–derived defense metabolites from plants: A potential source to facilitate novel antimicrobial development. J Biol Chem. 2021;296:100438. 33610552 10.1016/j.jbc.2021.100438 PMC8024917

[63] Xu J-J, Fang X, Li C-Y, Yang L, Chen X-Y. General and specialized tyrosine metabolism pathways in plants. aBIOTECH. 2020;1:97–105. 36304719 10.1007/s42994-019-00006-w PMC9590561

[64] Meena M, et al. Regulation of L-proline biosynthesis, signal transduction, transport, accumulation and its vital role in plants during variable environmental conditions. Heliyon. 2019;5:e02952. 31872123 10.1016/j.heliyon.2019.e02952 PMC6909094

[65] Ferrari S et al. Oligogalacturonides: plant damage-associated molecular patterns and regulators of growth and development. 2013. Front. Plant Sci. 4. 10.3389/fpls.2013.00049 PMC3595604 23493833

[66] Savatin DV, Gramegna G, Modesti V, Cervone F. Wounding in the plant tissue: the defense of a dangerous passage. 2014. Front Plant Sci 5. 10.3389/fpls.2014.00470 PMC4165286 25278948

[67] Rossell D, Zwiernik P. Dependence in elliptical partial correlation graphs. 2021. Electron J Stat 15.

[68] Xia X, Li J. Copula-based Partial Correlation Screening: a Joint and Robust Approach. Stat Sin. 2021. 10.5705/ss.202018.0219.

