# Supplementary figures and images for: Integrative multi-omics analysis reveals stress-specific molecular architectures in soybean under drought and rust infection
Source: BMC Genomics. 2026 Feb 28;27:348. doi: 10.1186/s12864-026-12673-3 (PMC13059611; doi:10.1186/s12864-026-12673-3)

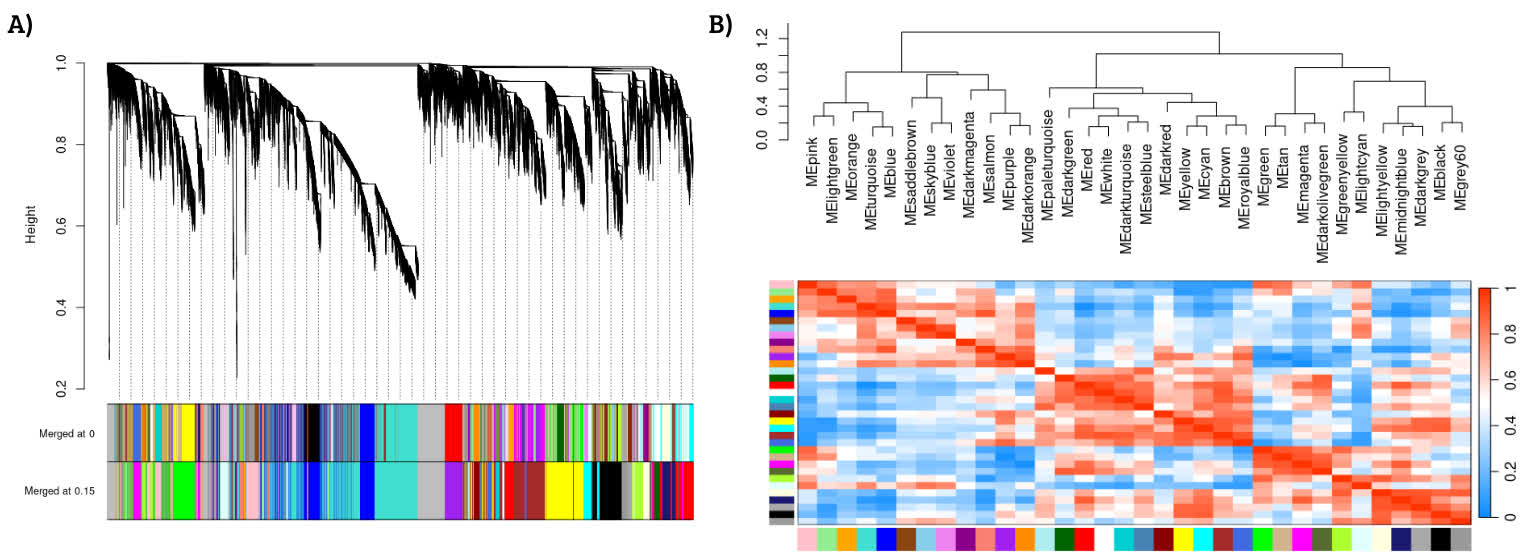

Supplement: Supplementary file 7 — Supplementary Material 7. [file 12864_2026_12673_MOESM7_ESM.jpg]

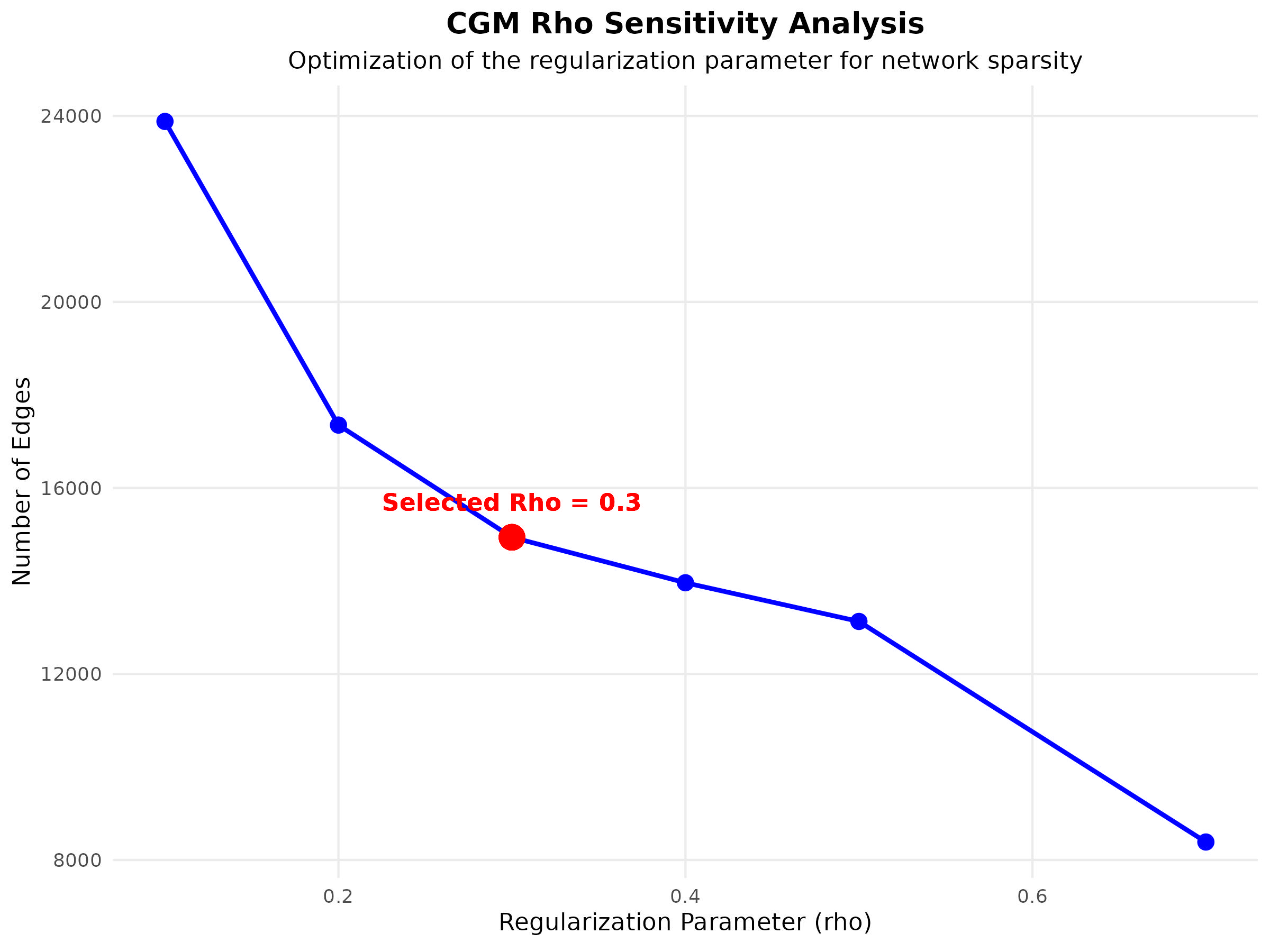

Supplement: Supplementary file 8 — Supplementary Material 8. [file 12864_2026_12673_MOESM8_ESM.jpg]
