# Supplementary material for: Integrative multi-omics analysis reveals stress-specific molecular architectures in soybean under drought and rust infection
Source: BMC Genomics. 2026 Feb 28;27:348. doi: 10.1186/s12864-026-12673-3 (PMC13059611; doi:10.1186/s12864-026-12673-3)
Supplement: Supplementary file 9 — Supplementary Material 9. [file 12864_2026_12673_MOESM9_ESM.pdf]

Supplementary data

Supplementary Table 1 - Hub persistence analysis of key gene co-expression modules across 50 bootstrap iterations.

Supplementary Table 2 - Resistance gene analogs (RGAs) identified in WGCNA module, including RGA family and subgroup classifications.

Supplementary Table 3 - KEGG pathway enrichment results for gene co-expression modules associated with metabolites from Groups 2, 3, and 5.

Supplementary Table 4 - List of differentially expressed genes (DEGs) including log2 fold change values across contrasts and time points.

Supplementary Table 5 - Partial correlation results for gene–metabolite pairs associated with fungal infection and water limitation.

Supplementary Table 6 - Edge-selection probability analysis for the CGM framework based on 50 bootstrap iterations.

Supplementary Figure 1 - Gene co-expression network construction and module identification using WGCNA. (A) Hierarchical clustering dendrogram of genes based on topological overlap, before and after module merging. Modules were initially defined using a dynamic tree cut method and subsequently merged based on eigengene correlation with a threshold of 0.15. (B) Heatmap and hierarchical clustering of module eigengenes showing the relationships among the 32 gene co-expression modules after merging. Module similarity is indicated by color intensity, with red representing high correlation and blue representing low correlation.

Supplementary Figure 2 - Elbow plot illustrating the relationship between the regularization parameter rho and the total number of edges in the CGM.
